# Modularized laparoscopic regional en bloc mesogastrium excision (rEME) based on membrane anatomy for distal gastric cancer

**DOI:** 10.1007/s00464-018-6375-x

**Published:** 2018-07-27

**Authors:** Jian Shen, Xiaogang Dong, Zhu Liu, Guoguang Wang, Jing Yang, Fei Zhou, Ming Lu, Xiang Ma, Yuan Li, Chaoyang Tang, Xiagang Luo, Qinghong Zhao, Jianping Zhang

**Affiliations:** 1 Department of General Surgery, The Second Affiliated Hospital, Nanjing Medical University, Jiangjiayuan No. 121, Nanjing, 210011 Jiangsu China; 2 The Jiangsu Key Lab of Cancer Biomarkers, Prevention and Treatment, Nanjing Medical University, Nanjing, 211166 Jiangsu China

**Keywords:** Gastric cancer, rEME, Membrane anatomy, D_2_ LN dissection, Surgical technique

## Abstract

**Background:**

The purpose of the study was to evaluate the safety and feasibility of a new surgical procedure named modularized laparoscopic regional En bloc mesogastrium excision (rEME) based on the membrane anatomy in distal laparoscopic radical gastrectomy for gastric cancer.

**Methods:**

From January 2014 to June 2017, 92 consecutive cases of patients with stages I–III distal gastric cancer were divided into 2 groups: laparoscopic radical gastrectomy plus standard D_2_ lymph node dissection (SD group, *n* = 44) and modularized rEME (rEME group, *n* = 48). Evaluations were made in terms of the operative data, pathological results, recovery time of digestive tract functions, complications, and length of stay.

**Results:**

85 patients (SD group, *n* = 40 and rEME group, *n* = 45) were finally included for analysis. There were no significant differences in the median total numbers of dissected LNs (31.98 ± 10.48 vs. 34.93 ± 13.12, *p* = 0.261), LNs in the greater curvature (12.18 ± 6.55 vs. 13.62 ± 8.09, *p* = 0.444), LNs in the lesser curvature (19.55 ± 7.40 vs. 17.98 ± 8.31, *p* = 0.365) between the SD and rEME groups. The rEME group showed lower loss of blood volume (107.11 ± 60.13 ml vs. 146.25 ± 85.78 ml, *p* = 0.019). No significant differences were found in recovery time of digestive tract functions, postoperative complication rates and length of hospital stay between the two groups.

**Conclusion:**

Laparoscopic radical gastrectomy plus modularized rEME based on the membrane anatomy is a safe and feasible procedure for distal gastric cancer.

Gastric cancer is one of the most common malignant tumors and the third leading cause of cancer-related mortality worldwide [1]. In general, incidence rates are highest in East Asia, including China [2]. In most countries, more than 80% of patients with gastric cancer are diagnosed with advanced gastric cancer (AGC). R0 resection combined with D_2_ LN dissection is recognized as the golden standard of surgical treatment for AGC especially in Asian countries [3]. In the past decade, laparoscopic surgery has become more widely accepted as a surgical treatment for gastric cancer (GC) because of its advantages in minimal invasiveness and also it has been increasingly applied in AGC [4–7]; however, laparoscopic D_2_ LN dissection is still technically difficult and its applicability is limited at present.

In the recent two decades, the total mesorectal excision (TME) [8] and complete mesocolic excision (CME) [9] with central vascular ligation (CVL) based on the embryology and anatomy has been recommended as the standard surgery for rectal and colon cancer, with excellent local control of the disease and improved survival rates. Different from TME and CME, en bloc mesogastrium excision (EME) seems impossible to perform attributable to the deficiency of a unified Toldt’s-like separating space nor can single vessel be ligated at the root. On the consideration of embryology and membrane anatomy, the pancreas is an exceptional organ which interrupted the extension of Toldt’s space to the upper abdomen, and all the inherent vessels of stomach origin from coeliac trunk are located in its surface. If we consider the pancreas as a landmark, the stomach’s mesentery can be divided into several relatively independent regions. In each region, total mesogastrium including LNs and vessels (right gastroepiploic artery RGEA, left gastroepiploic artery LGEA, right gastric artery RGA and left gastric artery LGA, respectively) can be resected en bloc.

Consequently, we present a modularized laparoscopic surgical procedure of rEME for distal gastric cancer and evaluate its safety and feasibility.

## Materials and methods

### Patients

We retrospective analyzed 92 consecutive patients who were performed laparoscopic radical distal gastrectomy in the Second Affiliated Hospital of Nanjing Medical University between January 2014 and June 2017. On the basis of previous studies [4–6], the inclusion criteria were as follows: age between 20 and 85 years old with pathological diagnosis of gastric cancer; *T*_1_, *T*_2 − 3_, or *T*_4a_ lesions and metastasis stage *M*_0_ pre-operation; a performance status of 0–2 according to the Eastern Cooperative Oncology Group (ECOG) [10]. Exclusion criteria: pathological *T*_4b_ tumors post-operation; previous treatment for cancer; previous upper abdominal surgery; emergency surgery (bleeding, obstruction, or perforation caused by gastric cancer). This study was reviewed and approved by the Ethics Committee of the Second Affiliated Hospital of Nanjing Medical University.

### Surgical technique

All patients were given general anesthesia and placed in the reverse Trendelenburg position with head elevated about 15°–20°. The surgeon stands on the patient’s left side, the assistant for camera stand between the patient’s legs, and another assistant is on the right side. The sequence of rEME was modularized as “infra-pyloric to supra-pyloric to supra-pancreatic to spleen hilar region” during the distal laparoscopic radical gastrectomy.

### Infra-pyloric region

**Fig. 1:**
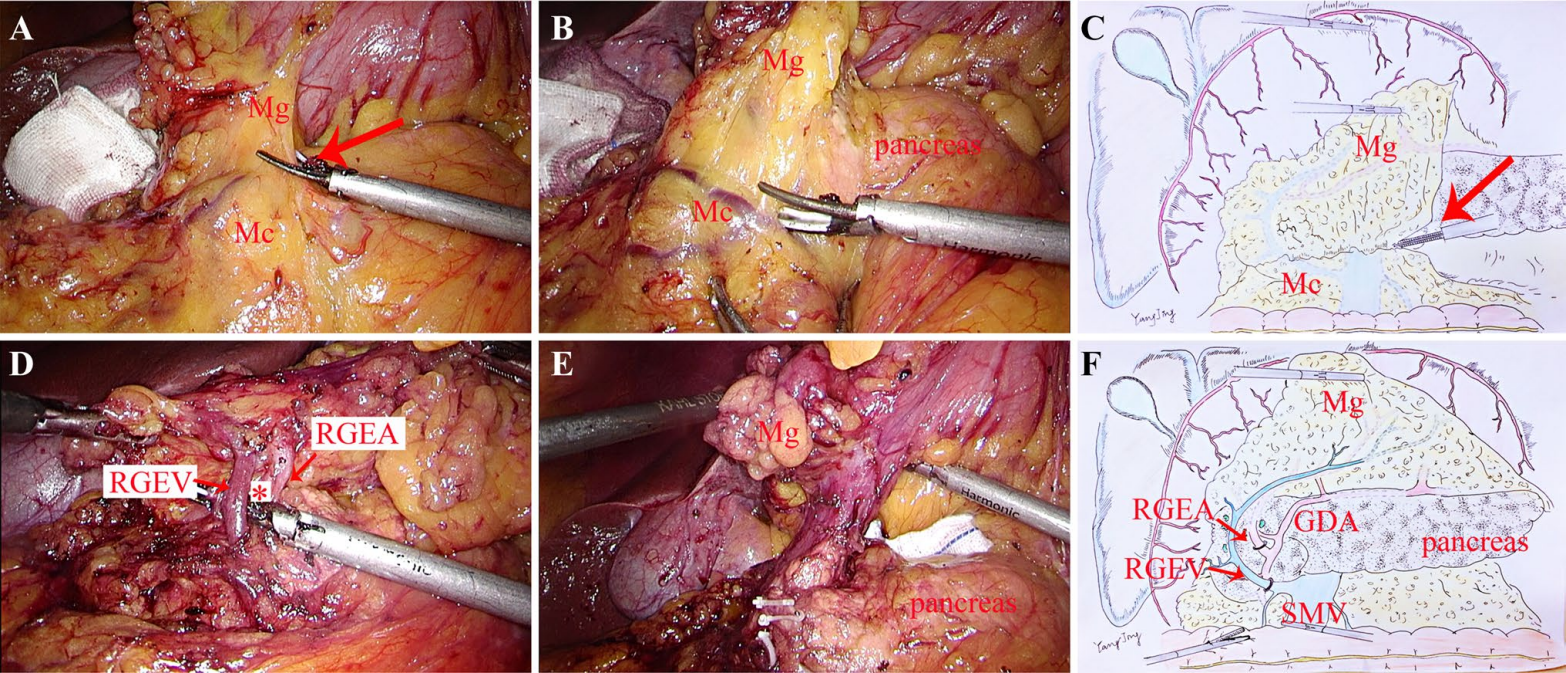
**A** Open the fusion gap of Mg and Mc (arrow). **B** The resection of Mg was begun under the pancreatic envelope. **C** The diagram of approach and separation space. **D** The RGEA and RGEV constitute a triangle together with the surface of pancreas (*). **E** The regional mesentery of duodenum and stomach were resected en bloc. **F** The diagram of rEME in the infra-pyloric region. (Color figure online)

*Approach and separation space* Firstly, the transverse mesocolon was detached from the mesogastrium (Mg) to follow the “line” of “inferior margin of pancreas.” Secondly, the pancreatic envelope was expected to open at the point of the pancreatic neck (projection of superior mesenteric vein, SMV) to the anterior pancreas space, and the separation continued following this space rightward (Fig. 1A, C). *Ligation of inherent vessels* The vessels, middle colonic vein (MCV), Henle’s trunk, accessory right colic vein (ARCV), right gastroepiploic vein (RGEV), right gastroepiploic artery (RGEA), and infra-pyloric artery IPA which distributed in this space were visible in sequence. The RGEV is commonly at the anterior of RGEA, and these two vessels together with the surface of pancreas constitute a triangle (we call it “right gastroepiploic golden triangle”) which was the landmark for ligating the vessels (Fig. 1B, D). By tracing the anterior pancreatic space, the lymphatic and adipose tissues were entirely dissociated from the duodenum (Fig. 1E). *Regional EME* When this procedure was correctly performed, the regional mesentery including No. 6 LNs was resected en bloc (Fig. 1F).

### Supra-pyloric region

**Fig. 2:**
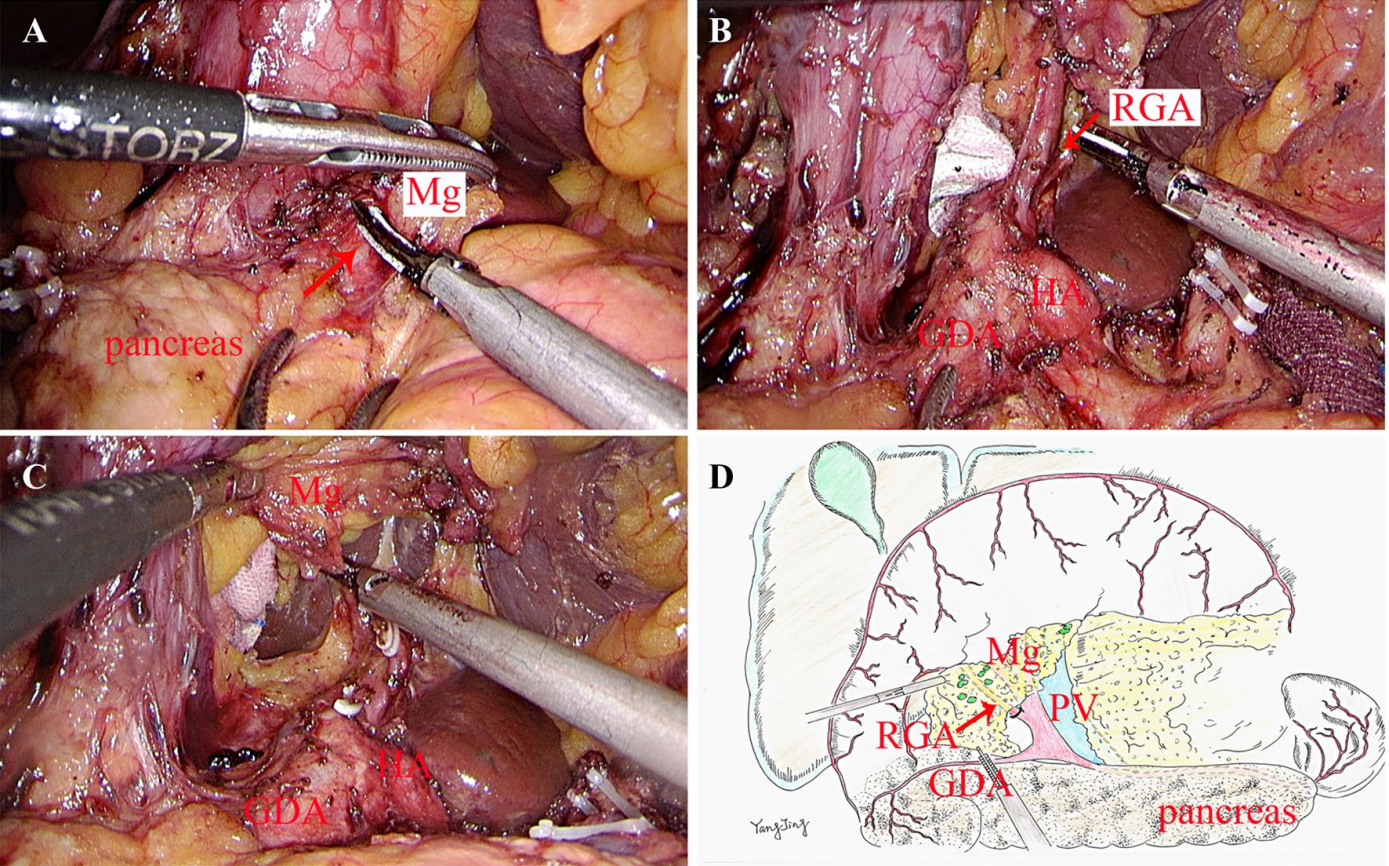
**A** The dissection of Mg was continued tracing the anterior pancreatic space (arrow) at the surface of GDA and CHA. **B** The total supra-pyloric mesentery was dissected tracing the surface of PHA upward to the hepatic hilum. **C** The RGA was released from the GDA and CHA and ligated at the root. **D** The diagram of rEME in the infra-pyloric region. (Color figure online)

*Approach and separation space* The separation of anterior pancreatic space continues following the gastroduodenal artery (GDA) (Fig. 2A). *Ligation of inherent vessels* The accessory mesentery of the RGA was released from the GDA and common hepatic artery (CHA), then the RGA was ligated at the root. The total supra-pyloric mesentery was dissected tracing the surface of proper hepatic artery (PHA) upward to the hepatic hilum with the common bile duct as right boundary and portal vein as left border (Fig. 2B, C). *Regional EME* The regional mesentery including No. 5, No. 12a, No. 12p and partial No. 8a LNs was then detached from the GDA and PHA and en bloc resected (Fig. 2D).

### Supra-pancreatic region

**Fig. 3:**
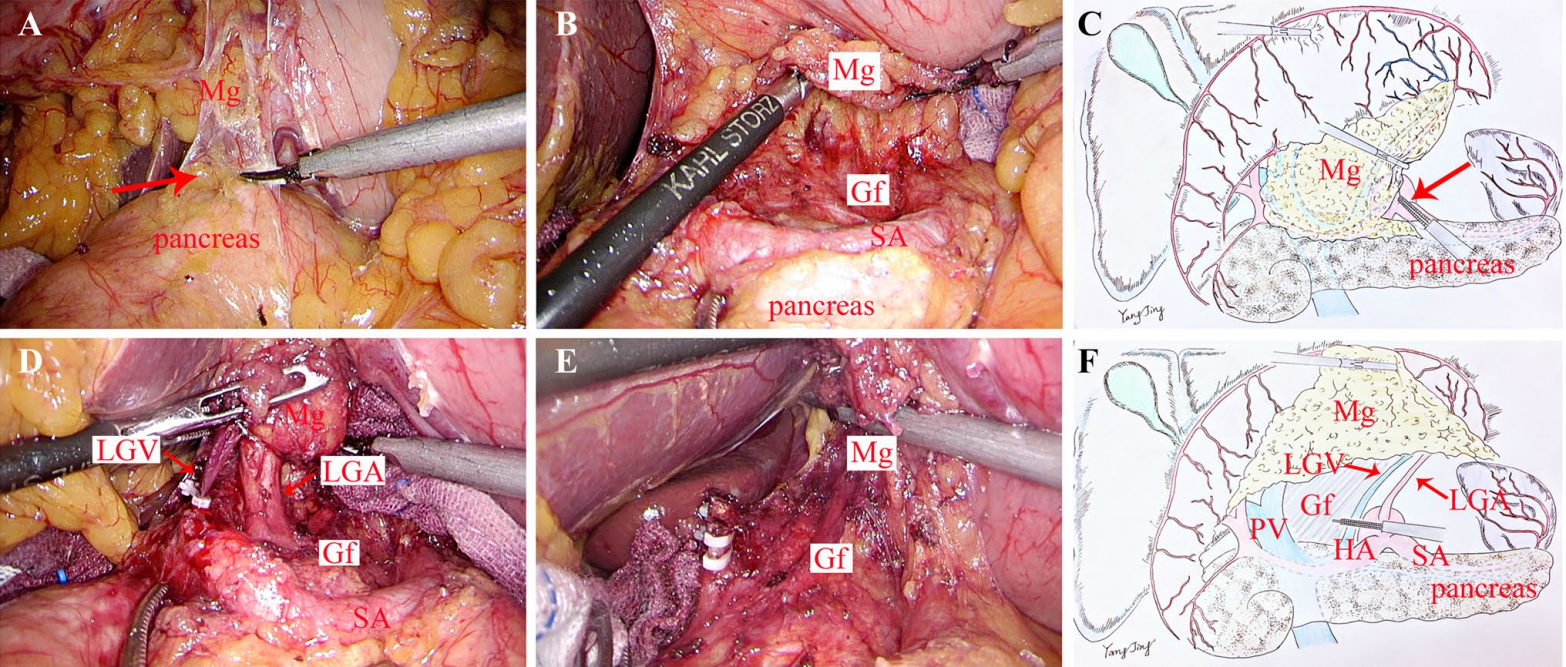
**A** The dissection began tracing the anterior pancreas space (arrow). **B** Dissect rearward following the surface of SA and expose the Gf. **C** The diagram of approach and separation space. **D** Dissection oriented by the Gf, and LGA and LGV were visible. **E** The LGA and LGV were both ligated at the root. **F** The diagram of rEME in the supra-pancreatic region. (Color figure online)

*Approach and separation space* The assistant holds the gastro-pancreatic plica with one endoscopic forceps and lift the pancreatic envelope with the other one. The surgeon started along the superior border of the pancreas to open the pancreatic envelope at the join of LGA and splenic artery (Fig. 3A, C), and then the dissection continued rearward to expose the posterior pancreas space with the landmark of Gerota’s fascia (Gf) (Fig. 3B). The Gerota’s fascia is embryologically the extension of Toldt’s space, and here exists a loose connective space lack of vessels between the mesogastrium and the Gerota’s fascia. *Ligation of inherent vessels* The dissection proceeds oriented by the Gerota’s fascia leftward till the root of the posterior gastric vessel (preserved) or the middle of splenic artery, and rightward until the left gastric vein(LGV), LGA were visible and ligated at the root in turn (Fig. 3D, E). The separation was continued rightward following the surface of CHA and finally achieved the dissection plane of supra-pyloric region subsequently. *Regional EME* By this step, the gastric mesentery in the supra-pancreatic region including No. 7, No. 8a, No. 9, and No. 11p LNs were en bloc excised. The dissection continued upward and leftward tracing the Gerota’s fascia till the wall of lower esophagus, and then the duodenum was cut off and the stump of stomach together with the mesentery of the lesser curvature were resected en bloc (Fig. 3F).

### Spleen hilar region

**Fig. 4:**
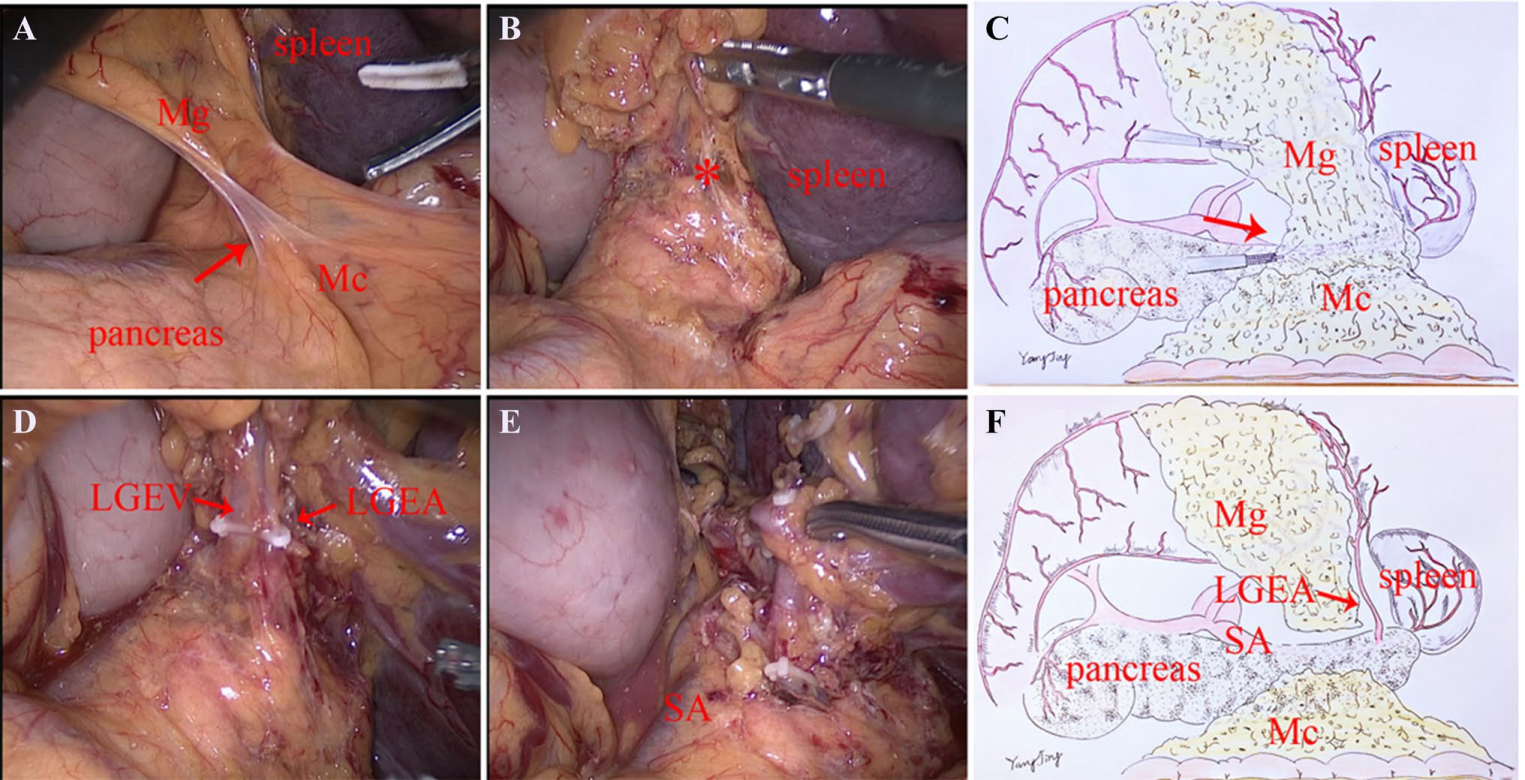
**A** The dissection began from the fusion plane of Mg and Mc (arrow). **B** Open the anterior pancreas space (*). **C** The diagram of approach and separation space. **D** The LGEV and LGEA were visible in turn and ligated at the root. **E** The separation was then turned to the wall of stomach. **F** The diagram of rEME in the supra-pancreatic region. (Color figure online)

*Approach and separation space* The assistant drafted the stomach rightward and the surgeon pulled the transverse colon oppositely to reveal the fusion plane of mesogastrium and mesocolon (Mc) (Fig. 4A, C). Then the pancreatic envelope was opened to expose the anterior pancreas space at the inferior border of the pancreas (Fig. 4B). *Ligation of inherent vessels* The end of the splenic vessels and LGEA were visible in turn by dissection tracing this plane. The mesentery containing No. 4sb LNs attached to LEGA was then pulled up and resected by ligation of the LEGA at the root (Fig. 4D). The separation was then turned to the wall of stomach. Two to three short gastric vessels with their mesentery including No4a LNs were excised by dividing at their roots (Fig. 4E). *Regional EME* The regional mesentery including No. 4sb, No. 4sa was then en bloc resected (Fig. 4F).

Finally, the distal gastrectomy and reconstruction of the gastrointestinal tract were performed.

#### Supplement

If it is necessary to expand dissection of splenic hilar lymph nodes, the complete mesentery in the splenic hilum containing No. 10 LNs should be en bloc resected following this plane according to the “Huang’s three-step maneuver” [11, 12].

The modularized rEME mentioned above can be successfully performed while avoiding disruption of mesogastrium and remnants of LNs by following these principles: un-touch of the tumor, correct surgical approach, accurate separating plane based on embryology and membrane anatomy, dissection tracing the plane instead of the vessels and en bloc mesogastrium resection.

### Statistical analysis

Consecutive variables were assessed via Student’s *t* test or *t’* test. Categorical variables were assessed via Pearson’s Chi-squared test or Fisher’s exact test. *p* values of less than 0.05 were considered to indicate statistical significance. The analyses were performed with IBM SPSS Statistics 19.0 (SPSS Inc., Chicago, IL, USA).

## Results

A total of 92 consecutive patients with preoperatively pathological diagnosed gastric cancer underwent laparoscopic distal gastrectomy plus radical lymphadenectomy at the Second Affiliated Hospital of Nanjing Medical University between January 2014 and June 2017. Patients were divided into two groups by different surgical procedures: laparoscopic radical gastrectomy plus standard D_2_ lymph node dissection (SD group, *n* = 44) and modularized rEME (rEME group, *n* = 48). Finally, 40 patients (25 males, 15 females; mean age, 63.25 ± 10.62 years) in SD group and 45 patients (29 males, 16 females; mean age, 62.02 ± 12.35 years) in rEME group were analyzed after excluded 7 *T*_4b_ patients.

We evaluated the operative date, complications, pathological results, recovery time of digestive tract functions, and length of hospital stay. Patient characteristics and operative outcomes are shown in Table 1. There was no significant difference in the mean longest diameter (3.14 ± 2.31 vs. 3.34 ± 1.87 cm, *p* = 0.666), *T*_1_/*T*_2_/*T*_3_/*T*_4a_ tumors (12/8/3/17 vs. 16/7/3/19, *p* = 0.981), LN metastasis rate (18/40 vs. 18/45, *p* = 0.631), patients with stage I, II, and III cancer (19/5/16 vs. 19/14/12, *p* = 0.110) between the SD group and rEME group according to the AJCC staging system. The average numbers of total harvested LNs (31.98 ± 10.48 vs. 34.93 ± 13.12, *p* = 0.261), LNs in the greater curvature (12.18 ± 6.55 vs. 13.62 ± 8.09, *p* = 0.444) and lesser curvature (19.55 ± 7.40 vs. 17.98 ± 8.31, *p* = 0.365) in SD group were similar to those in rEME group (Table 1). The rEME procedure required similar operative times (253.11 ± 55.52 vs. 265.63 ± 61.39 min, *p* = 0.333), but showed less blood loss during operation (107.11 ± 60.13 vs. 146.25 ± 85.78 ml, *p* = 0.019). One patient in each group was conversed to open surgery because of bleeding. All patients began to intake liquid the second day post-operation. The mean time of first flatus (3.35 ± 0.70 vs. 3.13 ± 0.98 days, *p* = 0.244), get out of bed post-operation (3.80 ± 2.14 vs. 4.35 ± 1.80 days, *p* = 0.200) and hospital stay (12.45 ± 6.53 vs. 11.44 ± 3.53 days, *p* = 0.374) in SD group were close to the rEME group. No incidence of hospital death was observed in the two groups. The overall incidence of postoperative complications was 20.0% in the SD group and 15.55% in the rEME group, with no statistically significant difference (*p* = 0.592) (Table 1).

**Table 1:** Patient characteristics and surgical outcomes

Variable	SD group	rEME group	*p* value
Number	40	45	
Age (mean ± SD)	63.25 ± 10.62	62.02 ± 12.35	0.629
Gender (male/female)	(25/15)	(29/16)	0.046
Longest diameter (mean ± SD)	3.14 ± 2.31 cm	3.34 ± 1.87 cm	0.666
Depth of invasion (*T*_1_/*T*_2_/*T*_3_/*T*_4a_)	(12/8/3/17)	(16/7/3/19)	0.981
Lymph node metastasis (*N*_0_/*N*_1_/*N*_2_/*N*_3_)	(22/3/6/9)	(27/6/4/8)	0.631
TNM stage (I/II/III)	(19/5/16)	(19/14/12)	0.110
Operation time, min (mean ± SD)	265.63 ± 61.39	253.11 ± 55.52	0.333
Blood loss, ml (mean ± SD)	146.25 ± 85.78	107.11 ± 60.13	0.019*
Number of dissected lymph nodes, number (mean ± SD)	31.98 ± 10.48	34.93 ± 13.12	0.261
Number of dissected lymph nodes in the greater curvature, number (mean ± SD)	12.18 ± 6.55	13.62 ± 8.09	0.444
Number of dissected lymph nodes in the lesser curvature, number (mean ± SD)	19.55 ± 7.40	17.98 ± 8.31	0.365
Postoperative hospital stays, days (mean ± SD)	12.45 ± 6.53	11.44 ± 3.53	0.374
The time to first flatus, days (mean ± SD)	3.35 ± 0.70	3.13 ± 0.98	0.244
The first time to get out of bed, days (mean ± SD)	3.80 ± 2.14	4.35 ± 1.80	0.200
Postoperative complications	8 (20.00%)	7 (15.55%)	0.592
Abdominal infection	1 (2.50%)	1 (2.22%)	1
Pulmonary infection	3 (7.50%)	4 (8.89%)	1
Anastomotic bleeding	1 (2.50%)	1 (2.22%)	1
Anastomotic leakage	1 (2.50%)	1 (2.22%)	1
Duodenal stump leakage	2 (5.0%)	0	0.219

Repeat cases not included

*p < 0.05

## Discussion

Gastric cancer is a common digestive tract malignant tumor with high incidence and mortality. Though the neoadjuvant chemoradiotherapy pre-operation accounts for increasing important role in the latest guidelines for the treatment of AGC in the West (including European Society of Medical Oncology, ESMO [13] and National Comprehensive Cancer Network, NCCN [14]). It is undeniable that the treatment of gastric cancer mainly relies on surgery both in the West and the East. Different from the West, D_2_ LN dissection plays a very important role or even as the golden standard of surgical treatment for AGC in the Asian countries including China [15]. Although every guideline for gastric cancer treatment emphasizes the importance of LNs dissection, but there is no standardized description for the details of the definition of LNs grouping and technicality for dissection. Most of the guidelines only require the harvested LN numbers over 15. The Japanese Gastric Cancer Treatment Guideline relatively characterizes the LNs grouping, but it is still impossible to standardize the details of the procedure [3]. In particular, LNs dissection during laparoscopic radical resection for gastric cancer is technically difficult along the blood vessels because of the various anatomic variations and the narrow visual field under laparoscopy. These factors attribute to the difference in the LNs dissection gastric cancer operations between different surgeons and hospitals. It will be helpful to improve the overall therapeutic results for gastric cancer if the procedure of LNs dissection can be relatively standardized. The aim of digestive tract cancer surgery is to resect the primary tumor with its accessory mesentery containing the lymphatic drainage system including sharp dissection of the visceral plane from the parietal plane which is the core technique in colorectal surgery of TME and CME. Briefly, intact layers of mesocolon was separated from the parietal plane and a maximum number of harvested LNs via true central ligation of the supplying vessels at their roots during TME or CME. The anatomical basis of this approach is that there exists an innate natural space between the mesocolon covered by an enveloped visceral layer and the retroperitoneal parietal plane which is called Toldt’s space [16, 17]. But it is not directly applicable for en bloc mesogastrium excision (EME) because the stomach has embryologically unique mesenteries compared with the colon. Bursectomy was one of the current surgical approaches while the key technical points of this procedure were to separate the anterior membrane of the transverse colon mesentery and the pancreatic capsule. It is technically difficult to perform bursectomy in laparoscopic surgery as surgeons would be prone to enter the wrong horizontal plane and injure the transverse colon vessels. On the other hand, the bursectomy was not improved the prognosis for patients with cT_3_(SS)/cT_4_a(SE) gastric cancer [18]. However, the principles (un-touch of the tumor, separating plane based on embryology and membrane anatomy, en bloc mesogastrium resection) in bursectomy by open were also crucial to obey in other laparoscopic surgical procedures for gastric cancer.

If we consider the pancreas, liver, and spleen as mesenteric components of the stomach, the Toldt’s space can be extended to the upper abdomen. The simplest way is to sharp dissection tracing the retroperitoneum parietal plane and ligated at the root of celiac trunk to completely remove the stomach and its mesentery. On the contrary, celiac trunk must be preserved as it offers arteries not only for stomach but also for other organs like liver, gallbladder, pancreas, and spleen. However, the four inherent arteries (RGEA, LGEA, RGA, and LGA) of the stomach origining from the celiac trunk can be detached and ligated at their roots. Moreover, the regional mesogastrium affiliated to these inherent vessels can be en bloc resected respectively. Accordingly, we divided the mesogastrium into four relatively independent regions to excision: infra-pyloric region, supra-pyloric region, spleen hilar region, and supra-pancreatic central region. There exists the constant fusion space between the mesogastrium and deep tissues for separation in their respective regions. These spaces can be considered as the cephalic extension of the Toldt’s space across the transverse mesocolon. The Toldt’s space was separated into two parts by the pancreas named anterior and posterior pancreas space, which fuse together again at the superior of pancreas and extend to the crura of diaphragm. The rEME can be easily performed tracing the anterior pancreas space between the envelope and pancreatic parenchyma in the infra-pyloric region, supra-pyloric region, and spleen hilar region (Figs. 1, 2, 4). The extending Toldt’s space in the supra-pancreatic central region is irregular due to the anatomical peculiarities, but the posterior part of the space named Gerota’s fascia is characterized as a smooth plane which can be easily recognized and oriented to complete rEME in this region (Fig. 3). Therefore, the ligation of vessels at the root and rEME can be successfully achieved according to reasonable surgical approach, correct anatomical space and modularized separation procedure in these four regions.

In most of the current studies, all regional LNs dissection is oriented by vessels. The most significant limitation of this approach is that the gastric mesentery will be broken during the sharp dissection of vascular and resulting remnants of mesenteric tissues containing LNs. While the mesogastrium could be resected en bloc by the rEME procedure which can avoid the tumor cell’s detachment and residue during operation and may obtain long-term survival benefits. Besides, it is always difficult to find the vessels directly in obese patients [19, 20]. It is believed that the contiguous mesogastric and fusion fascia can be separated by tracing mesothelial and connective tissue layers which generate the laparoscopic surgical planes for separation. The laparoscopic rEME oriented by mesofascial plane separation which is based on embryological considerations is easy to be modularized and it is helpful to avoid injury vascular and pancreatic parenchyma [21]. In this series, the mean intraoperative blood loss in rEME group was 107.11 ± 60.13 ml and only one patient conversed to open surgery because of bleeding which was better than the SD group. The length of hospital stay was 11.44 ± 3.53 days, which was similar to other studies from Korea and China [22–24]. The mean numbers of LNs harvested in rEME were 34.93 ± 13.12 which was more than the SD group (31.98 ± 10.48) but without statistical difference. This advantage may be revealed by a larger sample size in the future studies. In both groups, the mean numbers of LNs harvested were far exceeded the 15 LNs required by the guidelines [13, 14] and the average numbers of LNs in the greater curvature and lesser curvature were (12.18 ± 6.55 vs. 13.62 ± 8.09, *p* = 0.444) and (19.55 ± 7.40 vs. 17.98 ± 8.31, *p* = 0.365) in SD and rEME group, respectively. The quality of lymph node dissection can be maintained in comparison with that in previous reports whether from the West or the East [15, 25–27] which suggests that this procedure can provide pathologically reliable lymphadenectomy. By the anatomical sharp dissection tracing the reasonable plane and space, the average blood loss and duration of surgery were also similar to other studies from big centers [22, 25]. Postoperative complications developed in seven patients (15.55%), including Clavien–Dindo classification of IIIb in two. One of these two patients suffered anastomotic bleeding and leakage. The postoperative morbidity and mortality were similar to the SD group and the results in Chinese population [15, 22, 28]. This procedure was a modularized separation procedure following four principles: reasonable surgical approach, accurate anatomical space, vascular ligation at the root, en bloc mesogastrium resection. As a result, sufficient LNs were consistently retrieved by this procedure. Moreover, surgeons can learn to perform this modular procedure easily and quickly with a shorter learning curve. One limitation of this study is that the oncological outcomes were not mature, and the evaluation of the long-term outcomes is in progress.

## Conclusion

In conclusion, our standardized technique of modularized laparoscopic rEME for distal gastric cancer is safe and technically feasible. In order to determine the long-term clinical outcomes of rEME, longer period of follow-up and extension to multicenter of this procedure is ongoing.
